# A counselling intervention for individual strategies to prevent complications and strengthen resources during pregnancy in gynaecological care (AOK-Family +): study protocol for a cluster-randomised controlled trial

**DOI:** 10.1186/s13063-024-08215-5

**Published:** 2024-06-18

**Authors:** Mareike Krämer, Laura Wohlhüter, Lina Hermeling, Jan Koetsenruijter, Martina Kamradt, Michel Wensing, Manuela Bombana

**Affiliations:** 1 Department of General Practice and Health Service Research, University Hospital Heidelberg, Im Neuenheimer Feld 130.3, Heidelberg, 69120 Germany; 2 Department of Health Promotion, AOK Baden-Wuerttemberg, Presselstrasse 19, Stuttgart, 70191 Germany

**Keywords:** Pregnancy, Health knowledge, Health behaviours, Prevention, Health literacy

## Abstract

**Background:**

Lifestyle-related risk factors can increase complications during pregnancy and negatively impact the health of a mother and her child. Knowledge about these compliances among many pregnant women and women of childbearing age is lacking. In the study AOK-Family + , we propose the evaluation of a newly developed counselling intervention. The intervention aims to raise awareness and to provide relevant information about the impact of lifestyle-related risk factors during pregnancy. The aim of the proposed study is to evaluate the effect of this counselling intervention on women’s knowledge of lifestyle-related risk factors during pregnancy and the concomitant healthy behaviours.

**Methods:**

A cluster-randomised trial with three arms in Baden-Wuerttemberg, Germany, is proposed. Pregnant women and women of childbearing age will be allocated to one of three groups: online intervention, on-site intervention, or a waiting-list control. Trained counsellors from AOK Baden-Wuerttemberg, a German statutory health insurer, will conduct the counselling sessions. Data collection is conducted throughout validated questionnaires administered at three intervals: before counselling (*t*0), directly after counselling (*t*1), and at a 6-week follow-up (*t*2). The primary outcomes will be health knowledge and healthy behaviours relating to LRFFs during pregnancy. A process evaluation will examine the processes, used resources, and future implementations through additional quantitative questions and qualitative interviews and focus groups.

**Discussion:**

Based on this study, an implementation strategy for future conduction of lifestyle consultation during pregnancy could be developed with the aim of reducing pre- and post-mortem mobility and mortality.

**Trial registration:**

The German Clinical Trials Register DRKS00027804. Registered on 2022/01/12.

**Supplementary Information:**

The online version contains supplementary material available at 10.1186/s13063-024-08215-5.

## Background

### Background and rationale

Non-communicable diseases (NCDs) account for more than 60% of the global burden of disease [1]. In 2019, 41 million deaths worldwide were caused by NCDs [2]. They are the leading cause of death among women in Germany: 93.3% of all deaths among women are from NCDs, of which 40.7% are from cardiovascular disease [3]. According to the World Health Organization (WHO), these NCDs are linked to four modifiable risk factors: physical inactivity, an unbalanced diet, the consumption of tobacco, and the consumption of alcohol. The WHO defines these as lifestyle-related risk factors (LRRFs) [2]. In Germany, statutory health insurers are obligated to provide primary prevention services according to the law (§§20, 20a, 20b, 20c SGB V), including services dedicated to the prevention of disease and promotion of healthy behaviours in five key lifestyle areas: physical activity, nutrition, stress management, resource management, and substance use [4].

The unhealthy lifestyle behaviours people adopt are highlighted during particular life phases, such as pregnancy. Previous studies in Germany have shown that, for instance, 10.9% of mothers smoked during pregnancy [5] and 14% occasionally consumed alcohol [6]. Additionally, 21.9% of mothers were overweight at the beginning of pregnancy and 14.7% were obese [7]. A recent survey of pregnant women in Germany found that 31.8% did not know the adverse health effects of alcohol consumption during pregnancy. Comparably, 48.5% underestimated the negative effects of smoking tobacco during pregnancy. In addition, 70.8% did not know the composition of a healthy diet according to recommendations [8]. Interestingly, health care providers do not provide a standardised form of education regarding the impact of LRRFs on pregnancy and lactation [9]. Despite the evidence that inadequate health literacy is associated with smoking, a lack of conviction regarding medication, a lack of adherence, and increased risk perception, as well as the fact that strengthened health literacy can improve health and health behaviours during pregnancy, there are only a few routine health care programmes aimed at improving health literacy among pregnant women in Germany [10]. In line with this recognised research gap regarding the effects of counselling and information on birth-related topics, the National Health Goal “Health around Childbirth” calls for measures to improve the delivery of care with regard to LRRFs during pregnancy [11].

### Objectives

The AOK-Family + trial aims to evaluate the effects of online and on-site counselling interventions conducted at health centres by the statutory health insurer AOK Baden-Wuerttemberg. The intervention intends to raise the awareness of LRRFs and healthy behaviours relating to pregnancy, and its implementation in a counselling context as a service provided by a statutory health insurer has not, to our best knowledge, been tested before. The aim of the proposed study is to assess the effect of a lifestyle-oriented counselling intervention on pregnancy-related health literacy in terms of knowledge and healthy behaviours. In addition, the effects of the on-site and online interventions will be compared. The study will identify what effects, if any, occur. As the counselling context has not yet been tested as a service of the statutory health insurance and the intervention is being applied for the first time, the study has partly an exploratory character. Nevertheless, statistical testing was set up with a focus on the superiority of the intervention. Finally, the delivery of the interventions will be documented and evaluated. In addition, an accompanying process evaluation will be conducted in which client satisfaction, user-friendliness, practicability and the process flow will be assessed on the one hand, and the target group, the role perception of the counsellors as well as the positioning of the counselling within the German health care system will be examined on the other hand.

## Methods

### Study design and setting

A cluster-randomised longitudinal three-armed trial is proposed. Participants in the first study arm will receive an on-site counselling intervention, study arm two will receive an online counselling intervention, while those in study arm three comprise a waiting-list control group. Participants in the control group will be able to receive the consultation after data collection. The newly developed counselling intervention will address the relationship between LRRFs and pregnancy, covering information on nutrition, physical activity, relaxation, stress management, and substance use during pregnancy. This study will be accompanied by a process evaluation; further details are provided in the related sections. For the development of the study protocol, the standard protocol items of the recommendations for interventional trails (SPIRIT) [12] were used. Possible changes to the procedure will be communicated to all relevant groups such as investigators, funder, study registry and AOK Baden-Wuerttemberg employees. No audit of the trial is planned.

### Study population and counsellors

The target group will include pregnant women and women of childbearing age who are between 18 and 49 years, able to give consent and insured with AOK Baden-Wuerttemberg. The ability to read and understand German is a requirement for participation.

The study counsellors will be AOK Baden-Wuerttemberg employees who have completed a degree in nutrition or sports science and have experience with LRRF counselling. They will complete a standardised training in advance.

### Recruitment of study participants

Participants are made aware of the study with the help of flyers in waiting rooms of gynaecological practices. In addition, further advertisements will be placed via health insurance company advertising channels (e.g. social media and insurance magazines). Interested participants can then contact AOK Baden-Wuerttemberg by phone. After participating in the study, all participants will receive an incentive. The incentive is a voucher code for the e-book “Handbook of Family Health” [13] with a value of EUR 12,99.

### Randomisation process

**Fig. 1:**
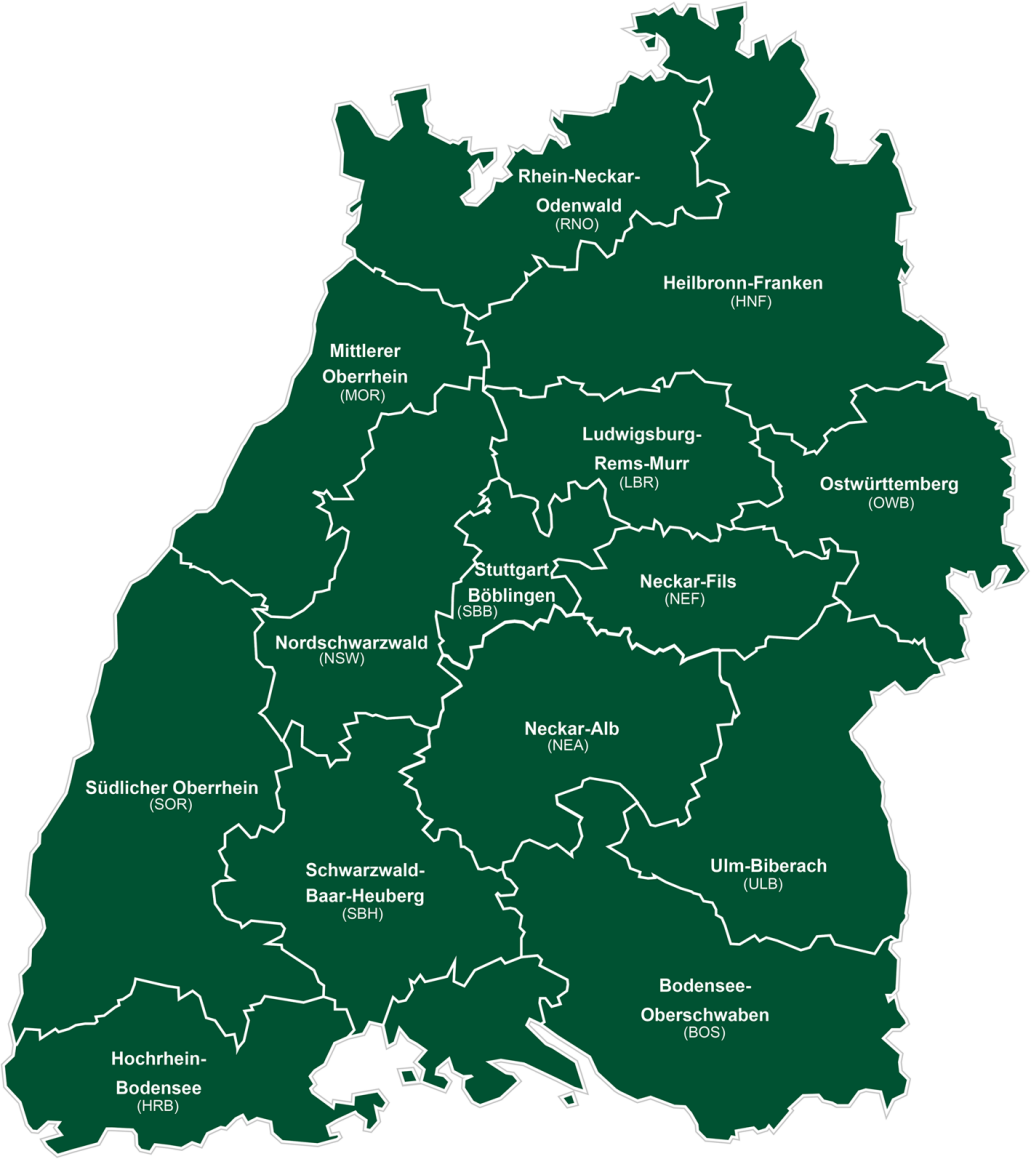
AOK Baden-Wuerttemberg district directorates

The 14 district directorates of AOK Baden-Wuerttemberg (Fig. 1), in Southwest Germany, are randomly assigned to one of the three study groups: the on-site intervention group, the online intervention group, or the control group. The initial randomisation process has been performed via computer generation and was carried out using a simple cluster-randomised design before the process of enrolment. The cluster randomisation will be conducted by an independent operator using the statistical program R (Version 1.3.5) with the randomizr package. The conduct of randomisation was concealed to the members of the study team. Similarly, the counsellors will be allocated according to their place of employment. Depending on where the insured people live, they will be assigned to the appropriate district directorate (e.g. if an insured person lives in the Ludwigsburg-Boeblingen area, she will be assigned to the Ludwigsburg-Boeblingen district directorate and the staff employed there). If an insured person is interested in the study, she will receive the intervention assigned to the responsible district directorate (e.g. insured person lives in the Ludwigsburg-Boeblingen area. The area has been assigned to intervention arm 1. The insured person is therefore automatically assigned to intervention arm 1). This means that the district directorates should be considered as randomised units.

### Assignment of interventions

Those interested in the study will be invited to contact AOK Baden-Wuerttemberg by phone to receive information on participation in the study from a trained staff member. As already mentioned, the participants were assigned to the intervention, which was randomly allocated to their district directorates. In the course of the telephone call, interested participants also receive information about the study arm to which their responsible district directorate has been assigned. Participants may stop the session at any point if they wish. There are no specific measures to increase adherence (e.g. to ensure participants attend and complete their session). Because this is a one-off session, the likelihood of patients stopping the session early was felt to be limited.

### Interventions

The interventions are educational, aiming to increase the awareness of LRRFs during pregnancy and the concomitant healthy behaviours supporting women. To achieve this, the interventions address LRRFs related to nutrition, physical activity, relaxation, stress management, and substance use for a planned period of 6 months. If in the course of counselling the need for further support services (e.g. nutrition counselling) is identified, the counsellor refers to the corresponding services. However, these are not part of the intervention. There were no restrictions on concomitant care. Overall, there is no blinding (including study participants) for the intervention. No possible harm data is collected. Termination of the study will be made by the study director. The SPIRIT [12] and TIDieR checklist [14] were used for the preparation of the intervention (see the Supplementary Materials). Table 1 shows the SPIRIT figure.

**Table 1:** Enrolment, intervention and assessments of the “AOK-Family + ”

	**Study period**				
	**Enrolment**	**Allocation**	**Post-allocation**		
**Timepoint**	*** − t***_**0**_	** − *t***_**0**_	**t**_0 *1_	***t***_1 *2_	***t***_2 *3_
**Enrolment:**					
** Eligibility screen**	X				
** Informed consent**	X				
** Allocation (cluster randomisation)**		X			
**Interventions:**					
*** On-site health counselling***				X	
*** Online health counselling***				X	
*** Waiting control group (does not receive counselling intervention)***					
**Assessments:**					
*** Health behaviour***			X		X
*** Health knowledge***			X	X	X
*** Customer satisfaction and usability***				X	X

^*^1 — should be answered up to 24 h before the counselling (control group – soon after receiving the questionnaire)

^*^2 — directly after the consultation (only for the intervention groups)

^*^3 — 6 weeks after *t*0

#### The three study arms

The three study arms are outlined in this section. Participants receiving the on-site intervention (arm one) will receive counselling for 45 min at a local health centre. The counsellors will use an information brochure on the impact of LRRFs during pregnancy and a conversation guide to support the process. Following this, the participants will be given the information brochure to take home.

In the online intervention (arm two), participants will engage with a 45-min online counselling session via a screensharing system with a video facility. The counsellors will share a digital PDF containing important information about the impact of LRRFs during pregnancy. The counselling process will also be supported by short videos of expert interviews (approx. 1 min per video), where relevant experts will provide information on the topics of nutrition, physical activity, relaxation, stress management, and substance use. Counsellors will receive a conversation guide to structure the counselling, and the participants will receive their information brochures via postal mail following the session.

Participants in the waiting-list control (arm three) will not receive counselling during the intervention period. However, at 6 weeks post-intervention, and after completing the *t*2-questionnaire, they will be invited to participate in online or on-site counselling and/or to receive the information brochure via mail.

All participants will have the option to terminate their participation in the study via a digital form on the AOK Baden-Wuerttemberg website. Table 2 summarises the three interventions.

**Table 2:** A summary of the three interventions of the study and the corresponding resource materials employed

On-Site Intervention, study arm 1	Online Intervention, study arm 2	Waiting-list Control, study arm 3
45-min on-site counselling at health centres covering the impact of LRRFs during pregnancy Materials • Information brochure provided for participants • Conversation guidelines provided for the counsellors	45-min online counselling covering the impact of LRRFs during pregnancy Materials • Interactive PDF shown via screen-sharing • Expert and explanatory videos • Information brochure mailed to participants • Conversation guidelines provided for the counsellors	No consultation about LRRFs Optional online or on-site consultation following full data collection (*t*2); the information brochure can be requested separately

#### Content of the counselling resources

Content of used resources for supporting participants and counsellors is based on the current scientific recommendations for nutrition, physical activity, relaxation, stress management, and substance use during pregnancy. This content was compiled from the guidelines published by relevant institutions and professional societies [15–17] and the current research evidence regarding LRRFs during pregnancy. These study resources were subsequently discussed and summarised by researchers at the Heidelberg University Hospital, Germany, and then prepared for inclusion in the counselling sessions. Several experts further reviewed and modified the content as necessary. This process is intended to ensure content consistency in all subject areas pertaining to the study and provides a valid platform for comparison of the three study groups. The design and finalisation of the resource materials were performed in collaboration with service providers associated with AOK Baden-Wuerttemberg.

#### Training for counsellors

Study counsellors must independently complete a 4-h e-learning module detailing the context and application of information on the study design and procedure, and the relevant scientific information on the relationship between LRRFs and pregnancy. The successful completion of the e-learning module requires an 80% minimum test score in a following multiple-choice assessment. Approximately 50 counsellors are expected to participate in the training. The e-learning module was developed by an external service provider associated with AOK Baden-Wuerttemberg.

Where medical questions and topics surpass the expertise and competencies of the counsellors, participants will be referred to the relevant care-pathway.

### Monitoring of the intervention process

Researchers from Heidelberg University Hospital will be available for consultation with the counsellors throughout the intervention period. In order to ascertain if the planned process will work as part of the counsellor’s daily routine, feedback will be sought from the counsellors after the initial health education sessions. If problems arise, the process, or the procedures, will be adjusted accordingly.

### Study outcomes

#### Primary outcomes

The study has two co-primary outcomes. The two primary outcomes of this study are health knowledge, and health-related behaviours, relating to LRRFs (nutrition, physical activity, relaxation and stress management, and substance use) during pregnancy.

Health Knowledge: Participants’ health knowledge related to LRRFs during pregnancy will be measured by a questionnaire developed by Oechsle et al. [8]. This questionnaire was developed to assess knowledge and attitudes of pregnant women concerning alcohol consumption, smoking, nutrition, physical activity, oral health, and medication. The questionnaire has been modified for the AOK-Family + study. In this process, an additional option (uncertain) was added to the previously dichotomous answer options (yes/no), and the individual wording of questions was adjusted. In addition, questions about oral health were replaced with questions about relaxation behaviours.

Health-Related Behaviours: Health-related behaviours have multifaceted domains. In this study, it will be assessed concerning nutrition, physical activity, relaxation and stress management, and substance use. As no all-encompassing instruments exist for this specific purpose, existing instruments for measuring health-related behaviours with regard to nutrition, physical activity, relaxation, stress management, and substance use were tested; those deemed suitable were adopted with a few adjustments according to the needs of the study. The final survey instrument is based on the AUDIT-C questionnaire, which is used to measure alcohol consumption [18, 19], and parts of the questionnaire from the study “German Health Update” (GEDA 2014/2015) by the Robert Koch Institute [20]. These parts relate to the assessment of physical activity (European Health Interview survey—Physical Activity Questionnaire (EHIS-PAQ)) [21] and tobacco use [20]. The Stress and Coping Inventory (SCI) [22] will be used to measure active stress management. Measurements on dietary behaviour were developed for this study. Changes in health behaviours are considered at the level of each category (physical activity, relaxation and stress management, and substance use).

##### Pilot testing and validation of the questionnaires

The questionnaires were piloted and validated in three consecutive steps. An expert interview was conducted with two midwives to verify the content and ensure the face validity of the questionnaire. Each topic in the questionnaire was discussed. The revised questionnaire was then iteratively piloted with seven women. The focus was on feasibility, comprehensibility and usability of the questionnaire. Finally, an empirical investigation was carried out in form of a cross-sectional study with 79 pregnant women. The plausibility of the results was assessed by reviewing the existing literature.

#### Secondary outcomes

##### Client satisfaction and user-friendliness

The secondary outcomes include client satisfaction, practicability and user-friendliness of the consultations. These will be evaluated using existing internal (not published) standard questions from AOK Baden-Wuerttemberg regarding customer satisfaction. The topics included are satisfaction with the enrolment process, satisfaction with the counsellor’s engagement, satisfaction with the counselling itself, possible recommendations for the counselling, and the subjectively perceived benefit of the counselling. This information is recorded using a 5-point Likert scale (strongly agree–strongly disagree). Mean values of the individual statements are then calculated and compared with previous internal results. These questions will only be addressed to the intervention groups (study arm one and two).

#### Other measures

All participants will be asked questions to determine their socio-demographic status. These include age, education, occupation, country of birth, parents’ countries of birth, and household income.

### Participant timeline, data collection, and management

Following verbal informed consent, a consultation appointment will be arranged for the intervention groups, and the participants will receive access (QR-Code and URL) to the first online questionnaire (*t*0), which will also be posted by mail. Verbal informed consent is recorded by the project team of AOK Baden-Wuerttemberg. Before starting the questionnaire, the participants’ consent is obtained again by actively ticking a checkbox. This baseline questionnaire should be answered up to 24 h before the counselling session (duration approximately 20–30 min) and will capture their socio-demographic data, health knowledge, and health-related behaviours. At the appointment, before the start of the counselling session, the participants will be asked whether the baseline questionnaire (*t*0) has already been completed. If not, completion of the questionnaire will be requested prior to counselling. Otherwise, the counselling intervention (on-site/digital) will take place and will last for approximately 45 min. Immediately after counselling, the counsellors will provide participants with access (QR-Code and URL) to the second online questionnaire (*t*1) to measure the short-term effect of the counselling on health knowledge, together with information regarding client satisfaction and the user-friendliness of the counselling (secondary outcomes) (duration 10 min). This questionnaire should be completed directly after counselling at the health centres or after leaving the digital room.

To measure the longer-term effects of the counselling, a follow-up questionnaire 6 weeks after *t*0 will be conducted, including participants’ health knowledge and healthy behaviours (*t*2), as well as their perceived user-friendliness and client satisfaction (duration approximately 30–35 min). The participants will receive access (QR-Code and URL) to the last online questionnaire by mail.

The waiting-list control group will receive access (QR-Code and URL) to the baseline questionnaire after they gave consent for study participation and receive the last questionnaire (*t*2) 6 weeks later by mail. After completion of the follow-up survey, the participants in the control group can individually arrange a requested AOK-Family + consultation. Table 3 summarises the timing and content of the questionnaires for each study arm.

**Table 3:** Summary of the timing and content of the questionnaires for each study arm (primary outcomes are highlighted)

	**On-site intervention, study arm 1**	**Online intervention, study arm 2**	**Waiting-list control, study arm 3**
***T*0 (posted by mail; answered up to 24 h before the counselling)**	Socio-demographic data, **health knowledge, health-related behaviours**	Socio-demographic data, **health knowledge, health-related behaviours**	Socio-demographic data, **health knowledge, health-related behaviours**
***T*1 (directly after counselling, access via QR-Code/URL)**	**Health knowledge**, client satisfaction user-friendliness of the counselling	**Health knowledge**, client satisfaction user-friendliness of the counselling	
***T*2 (posted by mail, 6 weeks after *t*0)**	**Health knowledge, healthy behaviours** user-friendliness, client satisfaction	**Health knowledge, healthy behaviours** user-friendliness, client satisfaction	**Health knowledge, healthy behaviours** user-friendliness, client satisfaction

All data will be collected anonymously using the online survey system LimeSurvey. The digital questionnaires can be filled out on personal smartphones or personal computers. Personal data, for identification as an AOK Baden-Wuerttemberg insured client, will be managed by AOK Baden-Wuerttemberg in accordance with their data protection standards. The Department of General Practice and Health Service Research (University Hospital Heidelberg) will only receive anonymous data from the questionnaires. The storage of this data occurs on a computer that is at least externally twofold encrypted and is protected against unauthorised access. Only researchers at Heidelberg University Hospital involved in the study have access to this data. A code created by the participants themselves at the beginning of each questionnaire enables the questionnaires from the different survey periods to be assigned accordingly. This code has five digits and is determined by four questions on invariable characteristics of the participants. Anonymity is preserved as the code remains exclusively with Heidelberg University Hospital and thus does not allow the person to be identified.

**Fig. 2:**
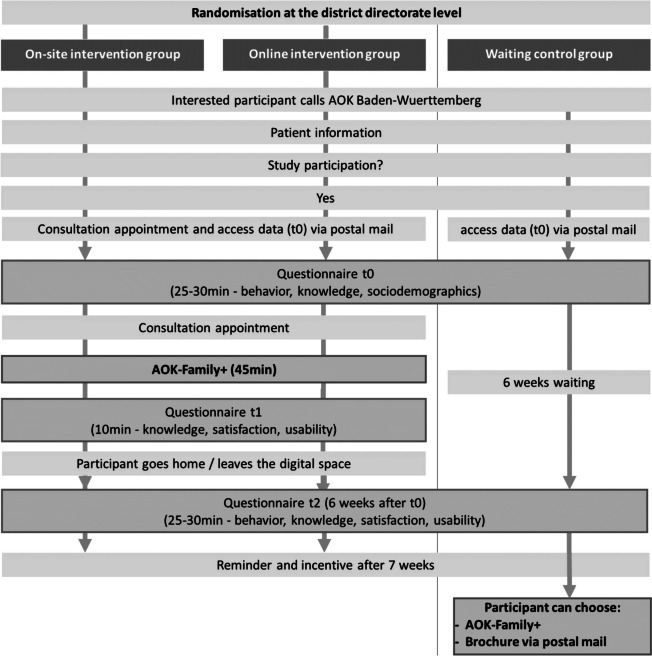
The study design with data collection and participant timelines

In order to generate robust data sets, the intervention will only start after the baseline questionnaire (*t*0) has been completed, and the second questionnaire will be directly administered during the appointment (*t*1). An independent person will be involved to monitor and check the integrity of data. To reduce the drop-out rate for the 6-week follow-up, participants will receive a reminder after 7 weeks and the incentive only after full participation. Participation is considered successful as soon as questionnaire *t*3 has been completed. This allows recording a change in health behaviour and health knowledge over a period of 6–7 weeks. Figure 2 illustrates the study design and process.

### Sample size calculation

The power calculation was performed assuming a probability of successful participation by 50% of participants in the control group, and a perceived relevant improvement of the successful participation by 65% of participants in the intervention groups. These assumptions were based on the results of a cross-sectional study by Oechsle et al. [8] on the health knowledge of pregnant women regarding LRRFs, in which knowledge gaps and their health effects were identified, and on studies indicating the need for improvement in the health behaviours of pregnant women [5–7, 23]. We calculated the sample size for the primary outcome “health knowledge” because for this outcome, we could derive a valid effect size from previous studies.

Furthermore, sample size calculations were based on a power of (1 − *ß*) = 65% and a significance level of *α* = 5% (two-sided).

To account for the cluster structure, with participants nested within customer and service centres, a small intra-cluster correlation coefficient (ICC) of 0.01 was assumed. As there are 14 district directorates of AOK Baden-Wuerttemberg (arm one = 4, arm two = 5, arm three = 5), it is estimated that there would be approximately 36 participants per cluster (cluster = district directorate). Based on these assumptions and the perceived relevant difference of 50% in the control versus 65% in the intervention group, the sample size per study arm will be *n* = 168. This implies a total sample size of *n* = 504. A dropout rate of 20% is assumed; therefore, *n* = 638 participants must be enrolled in the study at time *t*0 to reach *n* = 504 for analysis.

The above calculations were performed using R (Version 1.3.5) and the sjstats package (Version 0.18.1).

### Statistical analysis

A statistical analysis plan will be written, including the following aspects: Descriptive analyses will be conducted to determine the study population characteristics and the differences in the primary and secondary outcomes between the study arms. The primary analyses will be based on the intention-to-treat principle. Both intervention arms will be compared with the control arm. Additionally, a more in-depth analysis for each outcome separately will be provided. A multiple-imputation procedure will be used to handle missing data. No interim analyses are carried out beforehand. The results of the analysis will be publicised to all relevant groups through publications and reports. Access to the protocol is possible upon reasonable request.

#### Primary outcomes

To test the effect of the intervention on the primary outcomes (health behaviours and health knowledge) at *t*2, both intervention arms will be compared to the control arm as adjusted for baseline values (*t*0) for the primary outcomes using multivariate regression analysis with random coefficients for district directorates. This method offers the option to control for potential baseline differences between study arms.

The health knowledge survey is structured as a knowledge test. All correct answers are added up at the end to give a score. The variables are dichotomous outcome variables (i.e., in agreement (yes) or disagreement (no/uncertain) with recommendations). For each variable, there are a number of multiple response categories for which women have to select one or more correct answers. The “correct answers” are based on the existing guidelines and recommendations of the federal government. For further details please see Oechsle et al.

The assessment of each category according to health behaviours is based on the fulfilment of applicable health behaviour recommendations (fulfilment of recommendations yes/no).”

#### Secondary outcomes

Client satisfaction with and the user-friendliness of the consultations will be analysed accordingly without, however, controlling for baseline values, as these questions are directly related to the intervention and will only be asked in the intervention groups at *t*1 and *t*2. Possible associations with potential improvements in health knowledge and health behaviours will also be analysed using multivariate regression analysis. In addition, an exploratory analysis to determine which materials influence the effectiveness of the intervention and whether these differ according to the form of counselling (on-site/online) will be conducted.

#### Other exploratory analysis

To further analyse which participants experienced behavioural and knowledge changes after the intervention, exploratory analysis will be performed. This analysis will investigate what factors influence behavioural and knowledge change, e.g. socioeconomic parameters (education, income, occupational status, and socioeconomic status), the number of previous pregnancies, the number of biological children, and previous counselling regarding LRRFs.

## Process evaluation

The process evaluation will aim to document the integrity of the planned procedures and processes, as well as to examine resources used during the counselling (interactive PDF shown via screen sharing, expert and explanatory videos, and information brochure). In addition, appraisals of future implementations of counselling interventions in health care settings will be collected.

### Study population

Initially, all participants in the intervention groups (*n* = 426) will be asked additional questions about the materials provided in the consultation.

Furthermore, interviews (*n* = 20–25) and two focus groups (*n* = 7 each) with trained study counsellors from AOK Baden-Wuerttemberg will be conducted to assess procedures and processes. All counsellors will be recruited via a shared e-mail distribution list created specifically for the purposes of the project.

### Data collection and measures

Quantitative data will be used for the evaluation of the materials. At survey times *t*1 and *t*2, the intervention groups will be asked additional questions regarding the comprehensibility and scope of the materials, the application of these materials by the participants, and the perceived relevance of the information. In addition, the extent to which used materials complemented the counselling will be assessed. For this, self-developed questionnaires were used.

To obtain more information on future implementation, the group of pregnant women and women of childbearing age will be analysed beyond the socio-demographic data. Furthermore, the counsellors’ understanding of their role as well as the location of the counselling service in the German health care landscape will be evaluated within the interviews. In addition, the question of whether the counselling is a complementary or a substitute service will be investigated. Focus groups with counsellors will be conducted to evaluate the procedures and processes of the intervention (scheduling appointment, technical equipment, and counselling procedure), and their potential improvement. The interviews and focus groups will take place online or on-site in August and September 2023 and last 45 min and 2.5 h, respectively. Interview guidelines will be developed for this purpose.

Verbal and written informed consent will be obtained from all focus group and individual interview participants. As this is a pseudonymous procedure, consent forms are required to be signed. Alongside the data from the quantitative survey, the qualitative data will be stored at Heidelberg University Hospital.

### Data analysis

Initially, the evaluation will include a descriptive analysis of the quantitative data regarding the materials, followed by an evaluation of customer satisfaction. Furthermore, exploratory analysis will be conducted to be able to establish possible correlations between process flow and satisfaction.

The focus groups will be video-taped and transcribed verbatim, as protocols [24]. The individual interviews will be audio-recorded and then also transcribed verbatim. All qualitative data will be subsequently analysed with the help of qualitative content analysis methods [25, 26].

## Discussion

An adequate basic knowledge of nutrition, physical activity, relaxation, stress management, and substance use during pregnancy is fundamental for beneficial personal health care. Particular emphasis is needed on supporting women to remain informed and aware of the connection between a healthy pregnancy and the negative effects of addictive substance use, such as with alcohol and tobacco.

Overall, this study proposes a contribution to the individual efforts of pregnant women towards preventing the negative impacts of unhealthy lifestyle choices and may reduce lifestyle-related pregnancy complications and outcomes. Furthermore, the positive effects of LRRF counselling during pregnancy have an impact not only on pregnant woman and their children but, also, in the best-case scenario, on the entire family by encouraging family members to make positive behavioural changes. In addition, this study will examine which factors positively influence the effectiveness of counselling. It is conceivable that, in a further step, the application of the developed intervention and materials in routine gynaecological and obstetric care will be evaluated. Counselling interventions, offered by health insurers, to raise awareness of the impact of LRRFs during pregnancy are a novel area of research. The scientific evaluation of the effect of such offers could contribute to the implementation of high-quality and cost-effective counselling services in the long term.

This study foresees some limitations. At present, participation is only possible for AOK Baden-Wuerttemberg-insured clients, representing 4.6 million persons in Baden-Wuerttemberg. In addition, it will be difficult to prevent survey participants from accessing published information, rather than reporting what they currently know. This concern applies to both the intervention and control groups and may be partially accounted for by recording the survey response times and taking them into account when interpreting the results. It is realistic to expect that a certain proportion of respondents will rate their health behaviours more highly on paper, than actually occurs in their lived experience, which is a natural reflection of the human need for social acceptance.

This study can help achieve national health goals and promote the effective contribution of evidence-based information and counselling to an improvement in public health. However, further research will be necessary to determine whether improving public health knowledge and potentially preventing pregnancy complications will have a positive impact on the economy and reduce individual and/or public health costs.

## Trial status

This is protocol version 4, date: 11 June 2024 Recruitment began on the 24th of April 2023 and will conclude at June 30, 2024. The Interviews were conducted in autumn/winter 2023 and the focus groups is planned for July 26, 2024.

### Supplementary Information

Supplementary Material 1.

Supplementary Material 2.

## Data Availability

The dataset supporting the conclusions of this article is available from the authors but restrictions apply to the availability of these data, which were used under license from AOK Baden-Wuerttemberg (Germany) for the current study, and so are not publicly available. Data are, however, available from the authors upon reasonable request and with permission from AOK Baden-Wuerttemberg.

## Abbreviations

EHIS-PAQ: European Health Interview Survey-Physical Activity
ICC: Intra-cluster correlation coefficient
LRRFs: Lifestyle-related risk factors
NCDs: Non-communicable diseases
SCI: Stress and Coping Inventory
WHO: World Health Organization
